# eHealth Literacy and Adolescent Health in Japanese Female High School Students in Sendai: Cross-Sectional Study

**DOI:** 10.2196/73237

**Published:** 2025-06-30

**Authors:** Takashi Takeda, Kana Yoshimi, Sayaka Kai, Fumi Inoue

**Affiliations:** 1 Division of Women’s Health Research Institute of Traditional Asian Medicine Kindai University Osaka-Sayama Japan

**Keywords:** female adolescents, mental health, premenstrual symptoms, self-esteem, eHealth

## Abstract

**Background:**

In the digital age, adolescents increasingly rely on online sources for health-related information. eHealth literacy—defined as the ability to find, evaluate, and apply online health information—plays a crucial role in health outcomes. However, limited research exists on eHealth literacy among Japanese high school students, particularly on its association with menstrual health and psychological well-being.

**Objective:**

This study aimed to assess the eHealth literacy of Japanese female high school students and examine its association with premenstrual symptoms, psychological distress, loneliness, and self-esteem.

**Methods:**

A cross-sectional, web-based survey was conducted in December 2024 among 1607 female students from 2 public high schools in Sendai, Japan. A total of 909 students with regular menstrual cycles completed all survey items. The survey included the eHealth Literacy Scale (eHEALS), Premenstrual Symptoms Questionnaire, Kessler Psychological Distress Scale (K6), Revised UCLA Loneliness Scale, Rosenberg Self-Esteem Scale, and a numerical rating scale for menstrual pain. Statistical analyses, including Student t tests, chi-square tests, correlation analyses, and logistic regression analyses, were used to examine the relationships between eHealth literacy and various health outcomes.

**Results:**

The mean eHEALS score was 22.8 (SD 7.3), with 32.1% (292/909) of participants classified as having high eHealth literacy (eHEALS≥26). Students with higher eHealth literacy reported significantly lower levels of loneliness and higher self-esteem. The severity of premenstrual symptoms, particularly feeling overwhelmed, was significantly lower in the high eHealth literacy group. Additionally, interpersonal difficulties related to premenstrual symptoms were less prevalent among students with high eHealth literacy. Pearson correlation analysis indicated negative associations between the eHEALS score and psychological distress (K6) and loneliness, whereas a positive association was observed with self-esteem. Logistic regression analysis showed that high self-esteem was significantly associated with high eHealth literacy.

**Conclusions:**

This study highlights the importance of eHealth literacy in adolescent health care. Higher eHealth literacy is linked to lower levels of loneliness, higher self-esteem, and reduced premenstrual symptom severity, particularly feeling overwhelmed. Although the cross-sectional design limits causal conclusions, these findings suggest that higher eHealth literacy is associated with better mental and reproductive health in adolescents. Future research should adopt longitudinal designs, include more diverse populations—such as male adolescents—and explore additional contributing factors to better elucidate these associations.

## Introduction

### Growing Role of eHealth in Adolescent Health

In recent years, many people have begun to use eHealth information to manage their own health [1]. As internet accessibility continues to expand globally, more individuals are obtaining health-related information online. Internet use is also high in Japan, standing at 86.2% in 2023, according to data from the Ministry of Internal Affairs and Communications [2]. The usage rate is particularly high among adolescents aged 13 years to 19 years (98.7%). Adolescence is an important transition period from childhood to adulthood, during which adolescents undergo many physical, cognitive, and psychosocial changes; establish their ego; build their own world; and develop healthy lifestyle habits. During this period, adolescents seek information on topics such as sexual health, nutrition, beauty, and infectious diseases through websites and social media [3].

### Concept and Importance of eHealth Literacy

Health literacy is the ability to find, understand, and use health information to make informed decisions. These include reading medical instructions, understanding prescriptions, and evaluating health information from various sources. Good health literacy helps individuals manage diseases, correctly follow treatment, and effectively communicate with health care providers. It also involves numeracy skills such as understanding dosages and medical test results. Low health literacy can lead to poor health outcomes and medication errors. Therefore, promoting health literacy empowers individuals to take charge of their well-being and make healthier choices. Among digitally native adolescents, the internet has replaced traditional sources such as books for gathering health information [4,5].

### Assessment of eHealth Literacy Among Adolescents

Given this shift, eHealth literacy—the ability to find, evaluate, and use health information from the internet to solve one’s health problems—has become increasingly important [6]. The 6-item Lily Model of eHealth literacy, comprising traditional, health, information, scientific, media, and computer literacy, was previously proposed [6]. An 8-item rating scale called the eHealth Literacy Scale (eHEALS), based on this model, has been developed [7], translated into many languages, and used worldwide [8-11]. As adolescents continue to rely on the internet for health-related information, assessing their eHealth literacy is essential. However, despite increasing reliance on digital health information, research on eHealth literacy among adolescents remains scarce. Most studies have focused on university students or adults, leaving a significant knowledge gap regarding younger adolescents [4,12]. Moreover, global studies on adolescent eHealth literacy are sparse, with only a few investigations conducted in specific regions such as Turkey, Serbia, and Brazil [13-15]. These studies suggest variations in eHealth literacy levels among different populations, further highlighting the need for region-specific studies.

### Premenstrual Symptoms and Psychological Well-Being

Premenstrual symptoms are characterized by unpleasant psychosomatic symptoms that impair the quality of life of many women from adolescence to adulthood [16-18]. Premenstrual syndrome (PMS) is characterized by various premenstrual symptoms and is classified as premenstrual dysphoric disorder when psychological symptoms become particularly severe [19]. Recently, the concept of premenstrual disorders was introduced, considering both conditions as part of a continuum [20]. Menstrual pain and premenstrual symptoms are correlated and, as menstrual symptoms, have a significant impact on performance [21,22]. In Japan, a study with working women found that those with higher health literacy reported lower presenteeism and better performance at managing menstrual symptoms [22]. However, this study did not examine premenstrual symptoms or social dysfunction in detail. In addition, few studies have examined the relationship between health literacy, including eHealth literacy, and menstrual symptoms in adolescents.

### Psychological Distress, Loneliness, and Self-Esteem in Adolescents

High school students are at an increased risk of psychological distress, depression, anxiety, loneliness, and trauma [23]. Loneliness, the subjective feeling of isolation, has gained attention as a major public health concern owing to its strong association with mental health conditions such as depression and anxiety. It is also a key risk factor for poor mental health outcomes, particularly during adolescence [24-26]. A survey of Japanese high school students conducted in 2021 found that psychological distress was correlated with the severity of premenstrual symptoms, whereas loneliness was independently linked to both conditions. Self-esteem, which reflects an individual’s overall sense of self-worth [27], is another critical factor affecting the mental health of adolescents. Low self-esteem is linked to depression, eating disorders, risky behaviors, and academic decline [28]. Additionally, it has a strong reciprocal relationship with loneliness [27,28]. Low self-esteem and loneliness are particularly concerning during adolescence, which is the formative period for developing self-identity [29]. As adolescents increasingly rely on online health information, eHealth literacy may play a role in their psychological well-being by influencing their access to and interpretation of health-related content.

### Study Objectives

The purpose of this study was to assess the current level of eHealth literacy among Japanese female high school students, with a particular focus on examining its association with premenstrual symptoms. In addition, we investigated how eHealth literacy is related to other key aspects of adolescent health, including psychological distress, loneliness, and self-esteem. We hypothesized that students with higher eHealth literacy would report fewer premenstrual symptoms, lower levels of psychological distress and loneliness, and higher levels of self-esteem. These associations were examined to better understand the potential role of digital health literacy in promoting reproductive and mental well-being among adolescents.

## Methods

### Ethical Considerations

This study was conducted in accordance with the principles of the Declaration of Helsinki, and the Kindai University Ethics Committee approved the research protocol (approval number: R06-131; approval date: October 2, 2024). Students who participated in the study provided informed consent.

The Kindai University Ethics Committee approved the waiver of parental informed consent, and the students’ consent and willingness to participate were considered sufficient. The omission of parental informed consent was in accordance with the Ethical Guidelines for Medical and Health Sciences Research Involving Human Subjects issued by the Ministry of Education, Culture, Sports, Science, and Technology, and the Ministry of Health, Labour and Welfare. The data were anonymized and did not contain identifiable information regarding the participants. Participants were not compensated.

### Settings and Participants

**Figure 1:**
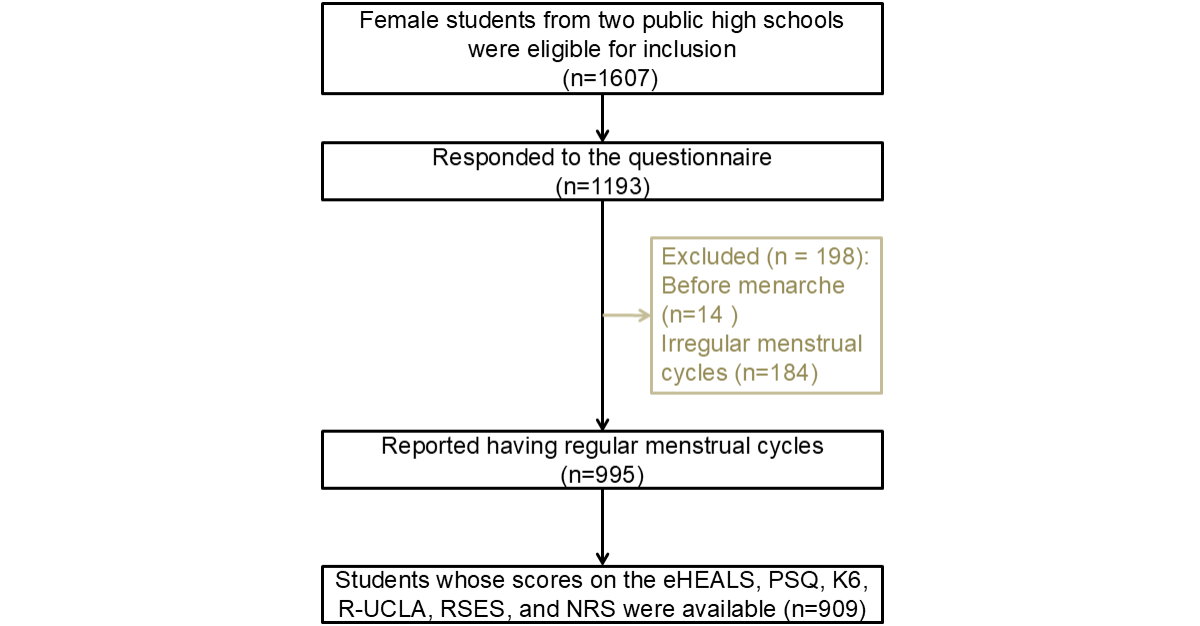
Study flowchart. eHEALS: eHealth Literacy Scale; K6: 6-item Kessler Psychological Distress Scale; NRS: numerical rating scale; PSQ: Premenstrual Symptoms Questionnaire; R-UCLA: 3-item Revised UCLA Loneliness Scale; RSES: Rosenberg Self-Esteem Scale.

In mid-December 2024, a school survey was conducted among 1607 female students attending 2 public high schools in Sendai, Japan. We conducted a web survey using Google Forms. The link to the self-administered survey form was distributed through school classes, and the survey was administered over the internet with consent. Given the sensitive nature of the questions, students were instructed to complete the survey independently at home after school hours, rather than during class time. A total of 1193 students responded to the survey. Of these, 995 had regular menstrual cycles of 25 days to 38 days (Figure 1). Because premenstrual symptoms appear solely during ovulatory cycles, only those with regular menstrual cycles were selected. Furthermore, 909 students who completed all items from the following measures were selected: eHEALS, Premenstrual Symptoms Questionnaire (PSQ), 6-item Kessler Psychological Distress Scale (K6), 3-item Revised UCLA Loneliness Scale (R-UCLA), Rosenberg Self-Esteem Scale (RSES), and numerical rating scale (NRS) for menstrual pain intensity.

### Questionnaires

#### eHEALS

The eHEALS is the only questionnaire that examines online health literacy [7]. In this study, we used the Japanese version, which has demonstrated validity and reliability [10]. The eHEALS is composed of 8 items and is scored on a 5-point scale (1=strongly disagree to 5=strongly agree). The total eHEALS score ranges from 8 to 40: the higher the score, the better the eHealth literacy. In this study, the Cronbach α coefficient for the scale was 0.935. Participants were divided into 2 groups—a high-scoring group (eHEALS≥26; n=292) and a low-scoring group (eHEALS<26; n=617)—according to criteria reported previously [29].

#### PSQ

The PSQ, previously used in research, was chosen for this study as a simple screening tool for premenstrual symptoms [18,30]. Its reliability and validity are well established [31]. The PSQ starts with: “In the past three months, have you experienced any premenstrual symptoms that start a week before menstruation and stop a few days after it begins?” It includes 11 items based on the Diagnostic and Statistical Manual of Mental Disorders–premenstrual dysphoric disorder criteria and assesses the impact of symptoms on (1) work and family responsibilities, (2) social activities, and (3) relationships. Severity and impact are rated on a 4-point scale (1=not at all to 4=severe). The total score (range: 14-56) indicates symptom severity. The Cronbach α was 0.936.

#### K6

The K6 was chosen for this study because it is a widely used screening tool for psychological distress in the general population [32,33]. Psychological distress was measured using the Japanese version of K6 [34], with established reliability and validity. The K6 consists of 6 items rated on a 5-point scale (0=not at all to 4=all). The total score ranges from 0 to 24, with higher scores indicating greater distress. The Cronbach α was 0.957.

#### R-UCLA

The R-UCLA was chosen for this study because it is a widely used and simple assessment tool for evaluating loneliness [35]. The Japanese version of the 3-item R-UCLA was used, and its reliability and validity have been well established [36]. This scale consists of 3 items rated on a 3-point scale (1=hardly ever, 2=sometimes, and 3=often), yielding a total score between 3 and 9. Higher scores indicate greater loneliness. The scale demonstrated strong internal consistency, with a Cronbach α of 0.913.

#### RSES

The RSES is a widely used self-esteem assessment tool that was selected for this study owing to its broad applicability [27]. The Japanese version of RSES (RSES-J) was used to measure self-esteem, and its reliability and validity have been confirmed [37]. The RSES-J consists of 10 items: 5 positive and 5 negative. Negative items are rated on a 4-point scale (1=strongly agree, 2=agree, 3=disagree, and 4=strongly disagree), whereas positive items are scored inversely. The total score ranges from 10 to 40, with higher scores indicating greater self-esteem. In this study, the Cronbach α for the RSES was 0.873.

#### NRS

Menstrual pain is closely linked to premenstrual symptoms [21,31]. In this study, pain severity was assessed using the NRS, a standard tool for measuring pain perception. Participants rate their pain on an 11-point scale ranging from 0 (no pain) to 10 (worst imaginable pain).

Participant data included age, school grade, weight, height, age at menarche, menstrual cycle length, internet usage time, and sleep duration. Menarche marks the onset of puberty and is linked to health issues such as early pregnancy, sexually transmitted diseases, depression, and anxiety [38,39]. It has been identified as a key factor in adolescent health. A regular cycle was defined as 25 days to 38 days. BMI was calculated by dividing weight by height squared (kg/m²). As obesity is associated with PMS, low self-esteem, and loneliness, BMI was included in the analysis [40,41].

### Statistical Analysis

The Cronbach α coefficient was used to assess the reliability of each scale (eHEALS, PSQ, K6, 3-item R-UCLA, and RSES). We calculated the means and SDs for continuous variables and proportions for categorical variables. Differences in the background characteristics of the high and low eHealth literacy groups were compared using Student *t* tests and Pearson chi-square tests. The Steel-Dwass test was used to compare differences in eHEALS scores between school years. The exact Wilcoxon signed-rank test was used to compare the severity of 11 premenstrual symptoms and 3 social disability items on the PSQ between the high and low eHealth literacy groups. Correlations among the 5 psychological questionnaires (total eHEALS, PSQ, K6, 3-item R-UCLA, and RSES) were examined using Pearson correlation coefficients. The factors significantly associated with high eHealth literacy were determined using multivariable logistic regression analysis. A total of 10 items—age, BMI, age at menarche, menstrual pain intensity, duration of internet use, sleep duration, total PSQ score, total K6 score, R-UCLA, and RSES—were included in the model.

Statistical analyses were performed using JMP Pro 18.1.0 (SAS). A *P* value<.05 was considered statistically significant.

## Results

### Participant Characteristics

The characteristics of all participants are presented in Table 1. The mean eHEALS score was 22.8 (SD 7.3). Approximately one-third (292/909, 32.1%) of the participants belonged to the high eHealth literacy group. The high eHealth literacy group had lower R-UCLA scores and higher RSES scores than the low eHealth literacy group.

**Table 1:** Characteristics of the study participants.

Characteristic		Overall (N=909)	Low eHealth literacy group (eHEALS^a^<26; n=617)	High eHealth literacy group (eHEALS≥26; n=292)	*P* value	
Age (years), mean (SD)		16.8 (0.9)	16.8 (0.9)	16.8 (1.0)	.88^b^	
**School year, n (%)**						.58^c^
	First year^d^	270 (29.7)	188 (69.6)	82 (30.3)		
	Second year^e^	297 (32.7)	195 (65.7)	102 (34.3)		
	Third year^f^	342 (37.6)	234 (68.4)	108 (31.6)		
BMI (kg/m^2^), mean (SD)		20.5 (2.6)	20.5 (0.1)	20.3 (0.15)	.29^b^	
Age at menarche (years), mean (SD)^g^		12.1 (1.3)	12.1 (0.1)	12.0 (0.1)	.12^b^	
Menstrual pain intensity, mean (SD)		4.8 (2.6)	4.9 (0.1)	4.8 (0.2)	.52^b^	
Duration of internet use (hours), mean (SD)^h^		3.1 (1.9)	3.2 (2.1)	3.1 (1.6)	.89^b^	
Sleep duration (hours), mean (SD)^i^		6.1 (1.0)	6.0 (1.0)	6.1 (1.0)	.12^b^	
Total PSQ^j^ score, mean (SD)		26.8 (9.5)	27.0 (0.4)	26.3 (0.6)	.24^b^	
K6^k^, mean (SD)		7.4 (7.4)	7.5 (0.3)	7.0 (0.4)	.27^b^	
R-UCLA^l^, mean (SD)		5.8 (2.7)	6.0 (0.1)	5.5 (0.2)	.02^b^	
RSES^m^, mean (SD)		25.1 (6.1)	24.3 (0.2)	26.8 (0.4)	<.001^b^	
eHEALS, mean (SD)		22.8 (7.3)	19.0 (5.2)	30.8 (4.1)	<.001^b^	

^a^eHEALS: eHealth Literacy Scale.

^b^Student *t* test.

^c^Pearson chi-square test.

^d^Median age: 16 (IQR 15-16) years.

^e^Median age: 17 (IQR 16-17) years.

^f^Median age: 18 (IQR 17-18) years.

^g^Missing data: 7 (0.8%).

^h^Missing data: 24 (2.6%).

^i^Missing data: 4 (0.4%).

^j^PSQ: Premenstrual Symptoms Questionnaire.

^k^K6: 6-item Kessler Psychological Distress Scale.

^l^R-UCLA: 3-item Revised UCLA Loneliness Scale.

^m^RSES: Rosenberg Self-Esteem Scale.

### Comparison of eHealth Literacy Between School Years

Differences in eHEALS scores between school years were analyzed in terms of the total score and each of the 8 items (Table 2). We found no differences in total eHEALS scores between school years. Of the 8 items, second-year students’ scores were significantly higher than those for first-year students for Q6 (I find on the Internet, I know how to use the Internet to answer my questions about health) and Q7 (I can distinguish high-quality health resources from low-quality health resources on the Internet). This suggests that some aspects of eHealth literacy may improve with age or school experience, but the overall literacy level remains consistent across the years.

**Table 2:** Study participants’ eHealth Literacy Scale (eHEALS) scores by school year level.

Question and total scores	First year, median (IQR)	Second year, median (IQR)	Third year, median (IQR)	*P* value (second vs first)^a^	*P* value (third vs first)^a^	*P* value (third vs second)^a^
Q1^b^	3 (2-3)	3 (2-4)	3 (2-4)	.48	.30	.90
Q2^c^	3 (2-3)	3 (2-3)	3 (2-3)	.35	.71	.82
Q3^d^	3 (2-4)	3 (2-4)	3 (2-4)	.41	.57	.97
Q4^e^	3 (2-4)	3 (2-4)	3 (2-4)	.54	.59	≥.99
Q5^f^	3 (2-4)	3 (2-4)	3 (2-4)	.40	.51	.98
Q6^g^	3 (2-3)	3 (2-3.5)	3 (2-4)	.02	.47	.33
Q7^h^	3 (2-3)	3 (2-4)	3 (2-4)	.02	.11	.82
Q8^i^	3 (2-3)	3 (2-4)	3 (2-4)	.08	.10	≥.99
Total eHEALS score	23 (16-26)	24 (19-28)	24 (18-28)	.10	.27	.88

^a^Steel-Dwass test.

^b^“I know what health resources are available on the Internet.”

^c^”I know where to find helpful health resources on the Internet.”

^d^”I know how to use the health information I find on the Internet to help me.”

^e^”I know how to find helpful health resources on the Internet.”

^f^”I have the skills I need to evaluate health resources.”

^g^”I know how to navigate the Internet and use it to answer my questions about health.”

^h^”I can distinguish high-quality health resources from low-quality health resources on the Internet.”

^i^”I feel confident in using information from the Internet to make health decisions.”

### eHealth Literacy and Premenstrual Symptoms

Next, the differences in premenstrual symptoms and level of social impairment were examined according to the level of eHealth literacy (high and low literacy; Table 3). Of the 11 premenstrual symptoms listed in the PSQ, only feeling overwhelmed was significantly lower in the high eHealth literacy group than in the low eHealth literacy group. In terms of social life impairment, only interpersonal impairment was significantly lower in the high eHealth literacy group than in the low eHealth literacy group. However, there were no significant differences in work productivity nor participation in social activities between the 2 groups.

**Table 3:** Differences by eHealth literacy level (high [n=292] and low [n=617] literacy) in terms of premenstrual symptoms and level of social impairment.

Characteristic				Responses to questions									*P* value^a^
				Not at all, n (%)		Mild, n (%)		Moderate, n (%)		Severe, n (%)			
**Premenstrual symptoms**													
	**Depressed mood**												.96
		Low eHealth literacy^b^	231 (37.4)		189 (30.6)		149 (24.2)		48 (7.8)				
		High eHealth literacy^c^	106 (36.3)		97 (33.2)		68 (23.3)		21 (7.2)				
	**Anxiety or tension**												.27
		Low eHealth literacy	166 (26.9)		211 (34.2)		183 (29.7)		57 (9.2)				
		High eHealth literacy	93 (31.9)		89 (30.5)		87 (29.8)		23 (7.9)				
	**Tearful**												.49
		Low eHealth literacy	244 (39.6)		159 (25.8)		151 (24.5)		63 (10.2)				
		High eHealth literacy	125 (42.8)		72 (24.7)		61 (20.9)		34 (11.6)				
	**Anger or irritability**												.10
		Low eHealth literacy	177 (28.7)		201 (32.6)		192 (31.1)		47 (7.6)				
		High eHealth literacy	99 (33.9)		95 (32.5)		75 (25.7)		23 (7.9)				
	**Decreased interest in work, home, or social activities**												.10
		Low eHealth literacy	333 (54)		164 (26.6)		95 (15.4)		25 (4.1)				
		High eHealth literacy	176 (60.3)		67 (23)		35 (12)		14 (4.8)				
	**Difficulty concentrating**												.41
		Low eHealth literacy	230 (37.3)		215 (34.9)		126 (20.4)		46 (7.5)				
		High eHealth literacy	118 (40.4)		96 (32.9)		59 (20.2)		19 (6.5)				
	**Fatigue or lack of energy**												.39
		Low eHealth literacy	159 (25.8)		198 (32.1)		183 (29.7)		77 (12.5)				
		High eHealth literacy	87 (29.8)		86 (29.5)		84 (28.8)		35 (12)				
	**Overeating or food cravings**												.32
		Low eHealth literacy	149 (24.2)		194 (31.4)		197(31.9)		77 (12.5)				
		High eHealth literacy	84 (28.8)		85 (29.1)		86 (29.5)		37 (12.7)				
	**Insomnia or hypersomnia**												.40
		Low eHealth literacy	233 (37.8)		157 (25.5)		148 (24)		79 (12.8)				
		High eHealth literacy	119(40.8)		72 (24.7)		66 (22.6)		35(12)				
	**Feeling overwhelmed**												.03
		Low eHealth literacy	343 (55.6)		172 (27.9)		78 (12.6)		24 (3.9)				
		High eHealth literacy	189 (64.7)		58 (19.9)		33 (11.3)		12 (4.1)				
	**Physical symptoms**												.86
		Low eHealth literacy	298 (48.3)		190 (30.8)		95 (15.4)		34 (5.5)				
		High eHealth literacy	144 (49.3)		77 (26.4)		56 (19.2)		15 (5.1)				
**Interference with work, usual activities, or relationships**													
	**Work efficiency, productivity, home responsibilities**												.55
		Low eHealth literacy	249 (40.4)		210 (34)		130 (21.1)		28 (4.5)				
		High eHealth literacy	128 (43.8)		90 (30.8)		56 (19.2)		18 (6.2)				
	**Social activities**												.15
		Low eHealth literacy	422 (68.4)		127 (20.6)		52 (8.4)		16 (2.6)				
		High eHealth literacy	214 (73.3)		50 (17.1)		22 (7.5)		6 (2.1)				
	**Relationships with coworkers and family**												.045
		Low eHealth literacy	431 (69.9)		142 (23)		33 (5.4)		11 (1.8)				
		High eHealth literacy	224 (76.7)		48 (16.4)		15 (5.1)		5 (1.7)				

^a^Exact Wilcoxon signed-rank test.

^b^eHealth Literacy Scale (eHEALS)<26.

^c^eHEALS≥26.

### Correlations Between eHealth Literacy and Psychological Measures

Subsequently, we examined the correlations between the eHEALS and the 4 psychological questionnaires used in this study (Table 4). The Pearson correlation coefficient analyses showed that the eHEALS score was negatively associated with the K6 total score (*r*=–0.071) and the R-UCLA score (*r*=–0.086) and positively associated with the RSES score (*r*=–0.170).

**Table 4:** Correlations among eHealth literacy, premenstrual symptoms, psychological distress, loneliness, and self-esteem.

Characteristics			eHEALS^a^		Total PSQ^b^		Total K6^c^		R-UCLA^d^		RSES^e^
**eHEALS**											
	*r*	—^f^		–0.023		–0.071		–0.086		0.170	
	*P* value	—		.49		.03		.01		<.001	
**Total PSQ**											
	*r*	–0.023		—		0.447		0.401		–0.358	
	*P* value	.49		—		<.001		<.001		<.001	
**Total K6**											
	*r*	–0.071		0.447		—		0.212		–0.285	
	*P* value	.03		<.001		—		<.001		<.001	
**R-UCLA**											
	*r*	–0.086		0.401		0.212		—		–0.468	
	*P* value	.01		<.001		<.001		—		<.001	
**RSES**											
	*r*	0.170		–0.358		–0.285		–0.468		—	
	*P* value	<.001		<.001		<.001		<.001		—	

^a^eHEALS: eHealth Literacy Scale.

^b^PSQ: Premenstrual Symptoms Questionnaire.

^c^K6: 6-item Kessler Psychological Distress Scale.

^d^R-UCLA: 3-item Revised UCLA Loneliness Scale.

^e^RSES: Rosenberg Self-Esteem Scale.

^f^Not applicable.

**Factors Associated With High eHealth Literacy**

We performed multivariable logistic regression analysis to identify the factors significantly associated with high eHealth literacy (Table 5) and found that high self-esteem was significantly associated with high eHealth literacy.

**Table 5:** Factors associated with high eHealth literacy (eHealth Literacy Scale≥26; R2=0.04).

Factors	Odds ratio (95% CI)	*P* value
Age (years)	1.026 (0.880-1.197)	.74
BMI (kg/m^2^)	0.954 (0.899-1.010)	.11
Age at menarche (years)	0.900 (0.801-1.091)	.07
Menstrual pain intensity	0.976 (0.917-1.040)	.46
Duration of internet use (hours)	1.015 (0.942-1.091)	.70
Sleeping duration (hours)	1.066 (0.929-1.224)	.36
Total PSQ^a^	1.010 (0.980-1.023)	.34
Total K6^b^	1.001 (0.979-1.023)	.94
R-UCLA^c^	0.996 (0.933-1.062)	.90
RSES^d^	1.076 (1.047-1.107)	<.001

^a^PSQ: Premenstrual Symptoms Questionnaire.

^b^K6: 6-item Kessler Psychological Distress Scale.

^c^R-UCLA: 3-item Revised UCLA Loneliness Scale.

^d^RSES: Rosenberg Self-Esteem Scale.

## Discussion

### Principal Findings

This study examined the eHealth literacy levels of female Japanese high school students and their associations with premenstrual symptoms, psychological distress, loneliness, and self-esteem. The findings highlight the importance of eHealth literacy in adolescent health and suggest that students with higher eHealth literacy tend to experience better mental and reproductive health outcomes. Additionally, this study contributes to the growing body of evidence indicating that digital health literacy plays a significant role in adolescent development and well-being—particularly as reflected in their health-related decision-making processes.

### eHealth Literacy Level

The results showed that the mean eHEALS score among participants was 22.8, with 32.1% classified as having high eHealth literacy. Compared with eHEALS scores of 23.6 and 23.5 for university students and the general population in Japan, respectively [10,12], the values obtained in this study appear to be slightly lower. As the data on adolescents overseas in the same age group are limited, performing comparisons becomes challenging. However, among 702 high school students in Serbia (mean age: 16.5 years), the average eHEALS score was 26.0 [14], and among 260 high school students in Brazil (mean age: 15.6 years), the average eHEALS score was 28.1 [15]. Based on these values, Japanese students appear to have a lower eHEALS score. The eHealth literacy of Japanese high school students obtained in this study was lower than that of Japanese university students, adults, and overseas high school students, suggesting a need for further improvement. The results of this study showed that eHEALS scores increased for some items in the second year compared with those in the first year, but there was no difference in scores between the years when assessed as a whole. Therefore, it is difficult to expect an improvement in eHealth literacy during the natural course of events, and educational interventions may be necessary.

### eHealth Literacy, Psychological Well-Being, and Social Connectedness

The negative association between eHealth literacy and loneliness suggests a potential link between digital health literacy and social connectivity. Adolescents with higher eHealth literacy may be more likely to engage with credible online health communities, which could help alleviate feelings of isolation. Additionally, the positive correlation between eHealth literacy and self-esteem indicates that adolescents with stronger digital health literacy may feel more empowered to make informed health decisions, potentially contributing to greater confidence and a more positive self-image.

### Association Between eHealth Literacy and Menstrual Symptom Severity

The intensity of menstrual pain and premenstrual symptoms, as assessed using the total PSQ score, did not differ according to the level of eHealth literacy. In terms of premenstrual symptoms, the high eHealth literacy group reported significantly lower levels of “feeling overwhelmed” than the low eHealth literacy group. However, other PMS symptoms, such as mood swings, fatigue, and irritability, were not significantly different between the groups. This suggests that, although higher eHealth literacy may be related to better psychological resilience, its direct relationship with overall premenstrual symptom severity remains unclear*.* Further investigation is needed to determine whether increased eHealth literacy leads to improved symptom management or whether other factors, such as coping strategies and social support, mediate this relationship. It is possible that individuals with high eHealth literacy possess more accurate knowledge of premenstrual symptoms, which might help them manage symptoms more calmly and avoid feeling overwhelmed, potentially reducing interpersonal difficulties. These findings suggest that higher eHealth literacy may be associated with improved psychological resilience and coping strategies for menstruation-related issues. Moreover, our results are consistent with those of prior research linking higher health literacy to improved management of menstrual symptoms among working women [22]. However, this study extends these findings by focusing on younger adolescents and exploring the relationship between eHealth literacy and premenstrual symptoms in relation to more detailed symptoms and details of social dysfunction, an area that has received less attention in previous studies.

### Correlation Between eHealth Literacy and Psychological Measures

The Pearson correlation analysis revealed a negative association between eHEALS scores and psychological distress (K6: *r*=–0.071; *P*=.03), as well as loneliness (R-UCLA: *r*=–0.086; *P*=.01), whereas a positive association was observed between the eHEALS score and self-esteem (RSES: *r*=0.170; *P*<.001). Although these associations were statistically significant, the effect sizes were small, suggesting that eHealth literacy alone may not be a strong predictor of psychological well-being. Additional factors, such as social relationships and coping mechanisms, should be considered in future research. These results suggest that adolescents with higher eHealth literacy are better equipped to access and interpret mental health information, which may help explain the lower psychological distress and higher self-worth observed in this study. Future research should investigate the causal relationships between eHealth literacy and mental well-being using longitudinal data.

### Factors Associated With High eHealth Literacy

The multivariate logistic regression analysis showed that high self-esteem was significantly associated with high eHealth literacy. These results align with previous findings among university students in the United Kingdom [42]. This finding highlights a strong link between self-esteem and eHealth literacy, suggesting that positive self-perception may be an important factor to consider in adolescent eHealth literacy programs.

### Limitations and Future Directions

Despite its strengths, this study has some limitations. First, the cross-sectional design precludes causal inference; therefore, longitudinal studies are needed to establish temporal relationships between eHealth literacy and health outcomes. Second, the study sample was limited to female students from 2 public high schools in Sendai, which may limit the generalizability of the findings to other regions and populations. Future studies should include more diverse samples, including students from different geographic and socioeconomic backgrounds. Expanding this research to include male adolescents and individuals from rural areas would provide a more comprehensive understanding of how eHealth literacy affects various demographics. Third, this study used self-administered online questionnaires completed by participants in their home environments. Although this approach offers convenience and accessibility, it has the following limitations: (1) potential response bias, including social desirability bias and misinterpretation of questions resulting from lack of direct supervision; (2) the home setting, which may have allowed outside influences or noncompliance with instructions, potentially affecting the authenticity of responses; and (3) collecting sensitive psychological and health-related information in an uncontrolled environment, which raises concerns about data integrity. However, discussing reproductive health is often taboo worldwide, especially in Asia, including Japan, with its conservative cultural and religious background [43]. Openly discussing menstrual issues in Japanese school classrooms is very difficult, and online after-school surveys are more likely to reveal the true status of students. Fourth, the study did not evaluate other participant-related factors that may influence eHealth literacy, such as parental education level, family economic status, and participants’ medical history [13,44-46]. Although the survey was administered online and completed by students after school, these questions were considered sensitive and difficult to ask. Therefore, it was challenging to include them in a study conducted as part of regular high school activities. Moreover, we acknowledge that other factors—such as access to internet resources, support from family and the community, and a family or maternal history of symptoms or gynecological or endocrine conditions—may also influence eHealth literacy and its related outcomes. Although these variables were not assessed in this study, their potential impact on health-related factors, such as self-esteem, should not be overlooked. These factors may interact with the psychosocial and reproductive health outcomes examined here. We recommend that future studies incorporate these elements to enhance the understanding of adolescent health in relation to eHealth literacy. Fifth, the logistic regression analysis in this study included 10 variables but did not account for potential confounders such as socioeconomic status, academic stress, home environment, and previous digital health education. As mentioned previously, items related to socioeconomic background and home environment were excluded because of ethical and practical limitations inherent in this school-based survey. Although past health education could have been included as a question, there are very few systematic digital health education programs for high school students in Japan. We judged that there was little variation in this factor and thus did not consider it meaningful as an analytical variable. Nevertheless, the omission of these factors may limit the comprehensiveness of the regression analysis, and future studies should include these variables as much as possible, depending on the circumstances.

Furthermore, this study focused only on specific health outcomes—namely, premenstrual symptoms, psychological distress, loneliness, and self-esteem. Future research should investigate additional physical and mental health outcomes to develop a more comprehensive understanding of how eHealth literacy influences adolescent health. Further research should explore potential interventions to enhance eHealth literacy and examine their long-term impact on adolescent health. In fact, a study with secondary school students in Spain demonstrated the educational impact of training sessions on eHealth literacy [47].

### Conclusions

In conclusion, this study highlights the importance of eHealth literacy for adolescent well-being. We found that higher eHealth literacy was associated with lower loneliness, higher self-esteem, and a reduced premenstrual symptom burden, particularly regarding feelings of being overwhelmed. These findings suggest that integrating eHealth literacy education into school programs could potentially benefit adolescents’ mental and reproductive health, although this should be confirmed in future studies. Future studies should aim to include more diverse populations—such as male adolescents and students from various socioeconomic backgrounds—and adopt longitudinal approaches to better understand the causal relationships between eHealth literacy and adolescent health outcomes. Future research should also focus on developing and evaluating interventions aimed at improving eHealth literacy and its long-term impact on adolescent health outcomes. By prioritizing digital health literacy initiatives, educators, health care professionals, and policymakers may help cultivate a more informed and health-conscious future generation.

## Abbreviations

eHEALS: eHealth Literacy Scale
K6: 6-item Kessler Psychological Distress Scale
NRS: numerical rating scale
OR: odds ratio
PMS: premenstrual syndrome
PSQ: Premenstrual Symptoms Questionnaire
R-UCLA: 3-item Revised UCLA Loneliness Scale
RSES: Rosenberg Self-Esteem Scale
RSES-J: Japanese version of the RSES

## Data Availability

The data obtained in this study are available from the corresponding author upon request.
